# Risk Characteristics of Peri-Implant Infections: A Retrospective Evaluation in a University Consultation Setting

**DOI:** 10.3390/dj10090159

**Published:** 2022-08-29

**Authors:** Achim V. Schwartzenberg, Chun Ching Liu, Philipp Sahrmann, Patrick R. Schmidlin, Ronald E. Jung, Nadja Naenni

**Affiliations:** 1 Careum Dental Hygiene, 8032 Zurich, Switzerland; 2 Clinic of Conservative and Preventive Dentistry, Center of Dental Medicine, University of Zurich, 8032 Zurich, Switzerland; 3 Clinic for Periodontology, Endodontology and Cariology, University Center of Dental Medicine Basel, 4058 Basel, Switzerland; 4 Clinic of Reconstructive Dentistry, Center of Dental Medicine, University of Zurich, 8032 Zurich, Switzerland

**Keywords:** peri-implantitis, risk characteristics, peri-implant mucositis, risk factors, periodontitis, systemic involvement, smoking

## Abstract

Peri-implantitis is a common biological complication in dentistry. The aim of the present study was to retrospectively analyze risk characteristics in a group of patients referred to a university-based consultation for peri-implantitis. In all, 190 initial cases from 2010 to 2019 were evaluated and descriptively summarized. The evaluation included various parameters such as periodontitis, smoking and oral hygiene status, implant position, type of prosthetic restoration and retention, mucosal quality, and further anamnestic and clinical findings related to the potential risk of developing peri-implantitis. Peri-implantitis was diagnosed in 83% of the cases, with peri-implant mucositis alone in 16% of cases; furthermore, 38% of the patients were diagnosed with active/instable periodontitis, while 14% had stable periodontitis. Residual cement was considered as a potential co-factor of peri-implant inflammation in 43% of cases. Suboptimal implant positioning was found in 19% of patients. Peri-implantitis or peri-implant mucositis was present in about one-third of patients in the absence of smoking and periodontitis factors. Of note, 6% showed no identifiable risk factors. Factors related to an increased risk of peri-implantitis should be taken into consideration when planning implant treatment. Adequate prosthetic implant position, restoration, and cleanability remain important for long-term success.

## 1. Introduction

Approximately 12–18 million dental implants are sold and placed worldwide each year [1]. Although implant survival rates are high, peri-implantitis and peri-implant mucositis are common biological complications [2]. The incidence of mucositis is reported to range from 19 to 65%, and peri-implantitis (PI) ranges from 1 to 47% [3,4,5]. With millions of dental implants sold and placed worldwide each year [6], when implant removal is necessary, peri-implantitis is the leading cause, followed by implant fractures [7]. Mucositis is diagnosed clinically via marginal signs of inflammation of the peri-implant mucosa without bone resorption, and the most important indicator is bleeding provoked by probing. In PI, there is also progressive resorption of the marginal bone around the implant, manifested by markedly increased probing depths and radiographic bone resorption. It is assumed that every case of PI is preceded by a basically reversible mucositis, which, similar to gingivitis and periodontitis, does not necessarily change into the irreversible form. 

A meta-analysis from 2018 [8] calculated the predictive power of bleeding on probing (BOP) in implants for PI. According to this study, 24% of all BOP-positive implants develop PI after one year or longer of functional loading. Another study found that the inflammatory response to biofilm around implants is more severe in teeth with gingivitis [5]. Accordingly, there is a higher potential risk of biofilm accumulation in implants due to suboptimal oral hygiene. According to Meyle and Chapple’s periodontitis development model [9], an ecological shift takes place in the microbiome of the oral biofilm, resulting in “dysbiosis”. This leads to chronic inflammation and bone resorption [10]. For this reason, similar to tooth-related measures, prophylactic and therapeutic measures are primarily aimed at reducing plaque by professional cleaning and optimizing individual oral hygiene. In addition, the host response remains an important factor. Common risk factors mentioned in the literature include a positive history of periodontitis, smoking, and various systemic diseases, such as osteoporosis and diabetes mellitus [11]. The Implant Disease Risk Assessment (IDRA) tool therefore lists a total of eight factors for peri-implant risk assessment [6]: BOP, probed pocket depth, prosthetic characteristics, bone loss of the most affected tooth in relation to age, general susceptibility to periodontitis, distance of the restoration margin to the bone, recall interval, and a history of periodontitis. Other influencing factors include the three-dimensional positioning of the implant in relation to the depth and position of the implant shoulder and the distance to neighboring implants or anatomical structures. In addition, there are prosthetic and (material) technical characteristics. 

Within this conceptual framework of risk factors and disease development, the present work aimed to assess typical risk factors in a regional population of patients referred to the interdisciplinary peri-implantitis consultation clinic at the University of Zurich. We hypothesized that the overall findings would be congruent with the available literature and that these factors are related to an increased risk of peri-implantitis.

## 2. Materials and Methods

**Figure 1:**
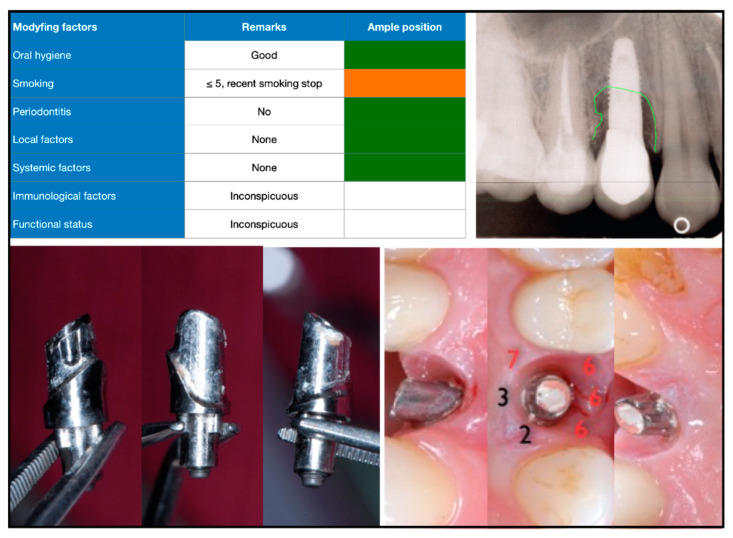
Initial findings in patient with healthy medical history (risk profile, top left). Tooth 14 was removed due to endodontic complication and ridge preservation was performed at that time. Three months later, implant placement was performed. Implant crown was cemented intraorally on an individualized abutment. Four years after placement, clear peri-implant bone resorption can be seen in radiograph (top right). Bottom left: removed abutment; bottom right: clinical findings of peri-implant mucosa with cement residue on abutment.

**Figure 2:**
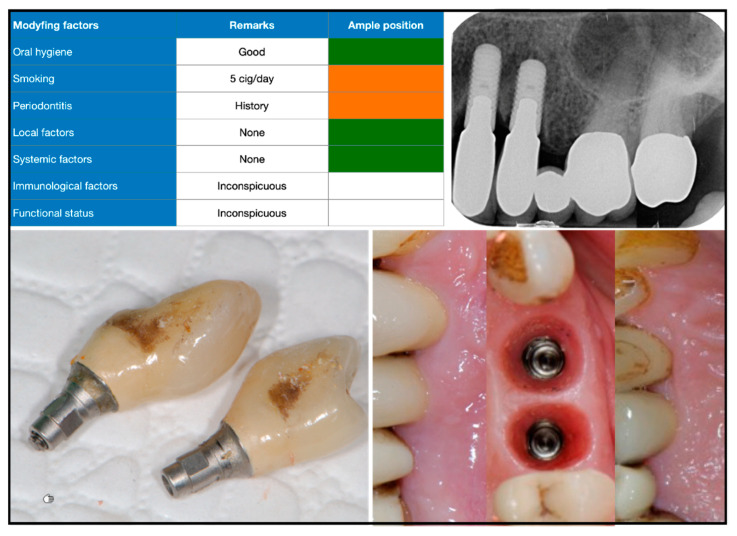
Initial findings in patient with history of periodontitis and smoking as co-factor (risk profile or traffic light, top left). Five years after implantation, recurrent abscesses formed at this site. X-ray image six years after operation shows vertical degradation patterns of peri-implant bone (top right).

**Figure 3:**
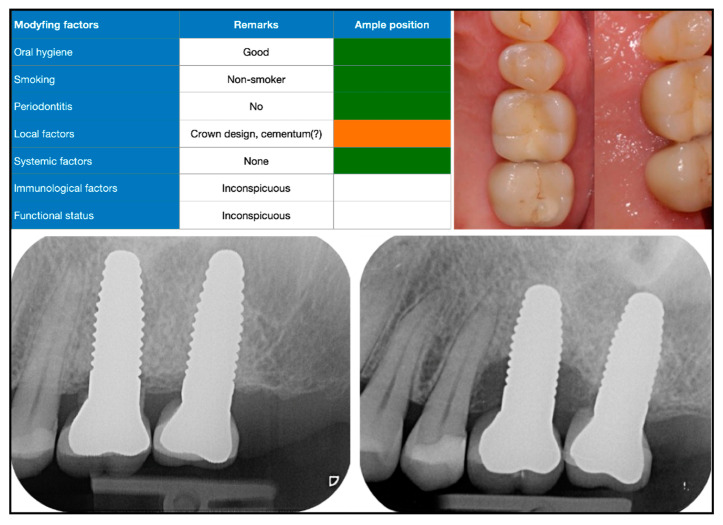
Initial findings in healthy patient with two implants in maxilla (risk profile or traffic light, top left). After healing, two crowns were cemented intraorally (initial radiograph, bottom left). Despite clinically and visually inconspicuous status, mesial implant showed rapidly progressive bone resorption a short time later (X-ray, bottom right).

We anonymized and digitally processed admission records from the peri-implantitis consultation clinic for further analysis. A total of 190 consecutive cases referred from 2010 to 2019 were available for data collection. The documentation contained the following information: medical history, referral (objective) and patient information/request (subjective), information on implant type, prosthetic restoration, insertion date, etiological factors, intraoral photographs, radiographs, periodontal parameters, diagnosis, and therapeutic suggestions. In addition, we employed a qualitative risk-oriented traffic-light scheme, which included case-related etiological aspects, such as oral hygiene, smoking status, and periodontal situation, and local, systemic, and functional factors (Figure 1, Figure 2 and Figure 3).

We transferred and saved this information in a data matrix (MS Excel 2019, Microsoft Corporation, Redmond, WA, USA) that contained 25 anamnestic and diagnostic parameters in addition to anonymized case IDs. If information was not provided, we set the corresponding variable to “NA” (not available). After data processing, we described and presented the distribution of parameters in tables and diagrams. We divided the results into the following main categories in the respective context: patients, prosthetics, and implant characteristics. 

## 3. Results

### 3.1. Patient Characteristics

A total of 688 dental implants in 190 patients were available for evaluation. The mean age of patients was 60 years (σ = 13.7 years), with an age range from 24 to 87 years. The proportion of women (N = 127) and men (N = 63) was 2:1. Among the patients, 173 were referred by external practitioners, and only 17 were self- or in-house referrals. Among external patients, 45% did not undergo implantation at the referrer’s site (implant placement alio loco), while 46% had implants placed by the referral clinician. Only 22% of patients (N = 41) reported pain, and 78% (N = 148) did not report any pain (Table 1). 

Systemic risk (e.g., diabetes and osteoporosis) was noted in 29% of patients; notably, 4% of patients in this group took bisphosphonate medication. Concomitant mental illness, such as depression, was present in 6%, as confirmed by a psychiatrist. Local factors (LFs) were considered as etiologically relevant in 46% of patients versus 49% without LFs. A sufficient band of keratinized mucosa, which was evaluated subjectively from photographs with no set measurements, was present in 67% of cases. In this patient cohort, 94% of cases mentioned risk factors, including local and systemic factors, i.e., prosthetic and biological factors, compared with 6% with no clear risk factors.

**Figure 4:**
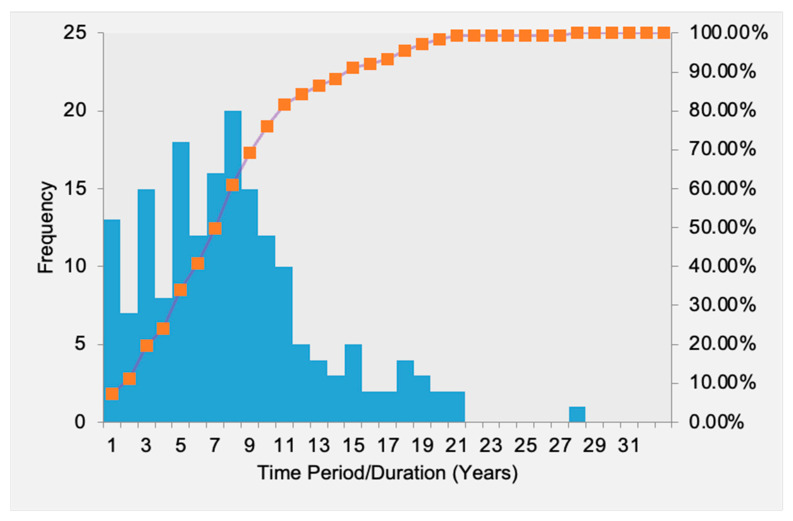
Time periods between implant placement and time of consultation. Blue bars indicate absolute number of patients, and line indicates cumulative percentages in corresponding time horizon (years; X-axis).

The time between implant placement and referral varied from 1 to 28 years, with a frequency peak at 8 years after implant placement (Figure 4). Eleven years after implant placement, the frequency of referrals dropped. 

**Figure 5:**
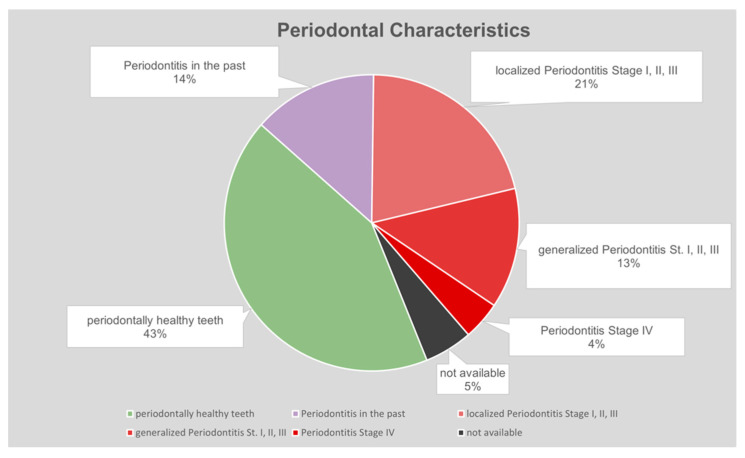
Distribution of periodontally healthy and diseased patients in sample. In 5% of cases, documentation related to this variable could not be evaluated.

**Figure 6:**
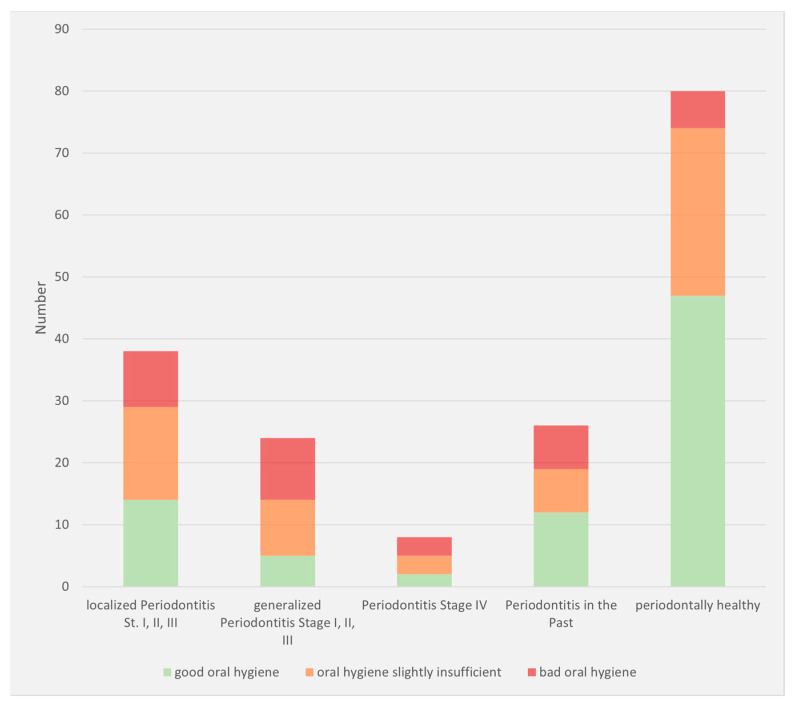
Oral hygiene status based on periodontitis manifestation (number: number of patients).

At the time of evaluation, 38% of patients had active periodontitis, and 14% had periodontitis in the past (Figure 5). Oral hygiene was rated as insufficient in 19%, in need of local improvement in 33%, and good to very good in 43% of cases (Figure 6).

**Figure 7:**
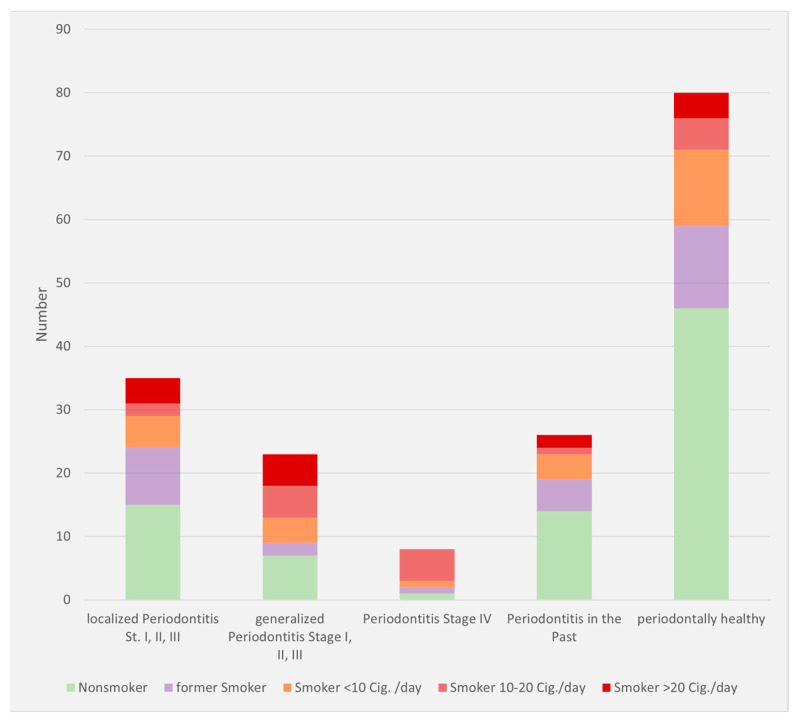
Smoking status according to patient data based on periodontitis manifestation (number: number of patients). Cig: cigarettes.

Smoking as a co-factor was present in 32% of patients; 16% percent were former smokers, and 44% reported never having smoked (Figure 7). Suspected parafunction was reported in 6% of cases. Additionally, 6% of patients had no identifiable risk factors for peri-implantitis.

### 3.2. Prosthetics 

In 77% of the cases, the suprastructure was easily cleansable by the patients. In 10%, cleansability was rated as moderate, and in 11% cleansability was difficult to impossible with regard to oral hygiene measures. Removable prostheses were found in 10% (cover denture prostheses supported by a bar, anchors, or telescopes). Fixed prostheses were found in 88% of the documented cases. Prosthetic reconstructions were screw-retained in 45% and cemented in 50%. Most prosthetic reconstructions were fabricated in the form of metal–ceramic restorations (82%). Radiographically, the transition between implant and abutment or prosthetic reconstruction was free of gaps and rated as sufficient in 82%. Significant marginal gaps or inadequate fit was found in 14% of cases (Table 2). Presumed cement-induced bone loss (Figure 2) was present in 7% of all cases, and cementation was considered a co-factor in 43% of peri-implantitis cases. 

### 3.3. Implant Distribution and Biological Complications 

The number of implants per patient varied from 1 implant (22%) to 16 implants (0.5%). A total of 388 out of the 688 implants evaluated were diagnosed as having a biological complication. Peri-implant mucositis was diagnosed in 31 patients (16%), and peri-implantitis was diagnosed in 158 patients (83%); furthermore, 49% of patients presented with peri-implantitis at one implant, and 51% of patients had more than one implant with peri-implantitis. The median probing depth measured was 5.5 mm (mean: 6 mm) (Table 3). Bone augmentation prior to or at the time of implant placement (guided bone regeneration or ridge augmentation) was documented in 21% (Figure 3). Sinus floor elevation had been performed in 8%. No information on the augmentation procedures was available for 45% of patients. Suboptimal, i.e., non-prosthetically driven, implant positioning was noted in 19% (optimal positioning: 80%). A radiographically visible pattern of bone resorption could be classified as horizontal in 44% of cases and vertical in 31% (no resorption was found in 20%) (Table 3).

## 4. Discussion

The present work retrospectively investigated the presence and distribution of individual risk characteristics in a specific patient population with peri-implant complications in a university-based consultation setting. A previously published retrospective study showed that healthy peri-implant ratios at 9–14 years post-implantation were predictive of future peri-implant health [12]. The positive benefit of regular recall sessions in the prevention of peri-implantitis (odds ratio (OR): 0.14) has also been demonstrated in studies [13]. Poor oral hygiene increases the risk of biofilm formation and, if predisposed, triggers an inflammatory response, leading to progressive bone resorption. In the investigated patient cohort, more than 80% of all biological complications occurred within the first 10 years after implant placement. This is in line with evidence suggesting that the onset of peri-implantitis occurs within the first 3 years of function and progresses over time [14,15]. The available literature clearly shows that the risk of developing peri-implantitis is increased (OR > 9) when there is pre-existing periodontitis [16]. After periodontitis is successfully treated, this can be reduced to approximately half [13]. The association of smoking with the development of periodontitis is reported in the literature, with an OR of 3 [8]. Regarding the development of peri-implantitis, the evidence in this regard is still weak. In a prospective study reporting on a 10-year period, approximately 20% of patients who smoked developed peri-implantitis; however only 6% of non-smokers developed the disease [17]. 

According to the consensus of the 2017 World Workshop on the Classification of Periodontal and Peri-Implant Diseases and Conditions, the evidence showing that a band of keratinized mucosa around implants was required was inconclusive, although it may be advantageous for patient comfort and ease of plaque removal [18]. However, a recent study concluded that a thin gingival phenotype and inadequate keratinized mucosa width (KMW < 2 mm) may be significant indicators for the risk of peri-implant disease [19].

An unfavorable design of the restoration can lead to biofilm accumulation and thus promote mucositis and peri-implantitis [20,21,22]. The contours of a restoration are related to the surgical position of the implant, restoration design, and aesthetics. Moreover, a recent cross-sectional study found that the presence of a convex profile and an emergence angle greater than 30° are significant indicators for the risk of peri-implantitis in bone-level implants [23]. 

In the case of cementation, biofilm can accumulate on excess cement. This could lead to peri-implant inflammation or host tissue reacting directly to the possible toxicity of the cement [24]. Wilson endoscopically assessed excess cement in a patient population and observed that removing cement remnants resulted in the healing/resolution of inflamed peri-implant tissues in 75% of the cases [25]. Peri-implant infection and cement excess were also found in the cohort of the present study. In patients with good oral hygiene and no risk factors, one might assume that cases of peri-implantitis might be cement-induced. Recent evidence suggests that zinc-based cement seems to inhibit biofilm bacterial growth in vitro, performs well clinically, and has favorable properties compared to resin-based cement [26,27]. Thus, the type of cement used appears to make a difference. In the present study, we lacked information about the cement type, which can be a limitation in this regard. Nevertheless, techniques to remove excess cement should be prioritized in combination with diagnostic methods to minimize the risk of residual excess cement [28,29]. A systematic review proves that biological complications occur more frequently in cemented reconstructions, while screw-retained reconstructions exhibit more technical complications, such as screw loosening and screw fracture [30]. Overall, screw-retained and -cemented reconstructions have their own advantages and disadvantages, with no significant differences in terms of survival rates [31,32]. 

The quality of the marginal seal in the area of the implant-abutment or abutment-restoration interface varies depending on the qualities of the transfer procedure and the dental technical work. Manufacturing-related gaps or steps may also have an influence on the development of peri-implantitis [20]. In the investigated group, the interface quality was insufficient in a total of 14%, i.e., gaps or steps were visible on radiographs. Gross positioning errors of implants leading to bone resorption [33] and inter-implant distances that were very small occurred only rarely in the investigated group. However, suboptimal implant positioning combined with other risk factors was observed in 19% of cases and may be a co-factor in the development of peri-implant infection. Data regarding implant systems and implant surface characteristics were not presented in this study due to the wide variety of systems from various companies. A recent review revealed good long-term survival rates independent of implant surface and roughness [34]. 

Certain genetic predispositions to peri-implantitis, such as tumor necrosis factor-alpha (TNF-⍺) or interleukin-1 (IL-1) polymorphisms, showed potential links in the onset of peri-implant diseases [35,36,37], although clear genetic patterns are still to be determined. In addition, immune-centered therapeutic approaches to improve osseointegration and prevent bone loss around implants were recently proposed in a review by Albrektsson et al., who emphasized the role of foreign body reaction and host response in the development of peri-implantitis [34]. These may account for the small percentage of patients with no clear risk factors identified in the present review.

The number of cases with bone augmentation procedures was reported in this study; however, it is important to bear in mind that hard and/soft tissue augmentation procedures do not increase the risk for biological complications, as concluded by a recent consensus report [38] based on a systematic review by Salvi et al. [39]. 

The following limitations must be considered due to the retrospective model of this study: Firstly, the treatment durations of the anti-resorptive medication (ARM), including bisphosphonates (BP), type of medication, and the time-point of implant placement, were not available for all patients. Although low-dose BP has not been shown to be a risk factor for peri-implant diseases based on current evidence, it is important to note that high-dose ARM could result in implant-related complications [40,41,42]. Additionally, data regarding the development of peri-implantitis in patients with a history of periodontitis were not available. 

In summary, all the risk factors described in the literature were present in the studied patient population. Some of the established factors, such as an ill-fitting suprastructure and the reconstruction not allowing for oral hygiene, should be avoided or corrected. Other factors, such as inadequate plaque control, require both informing and re-motivating patients and including them in a strict recall protocol. Ongoing periodontitis needs to be addressed prior to implant therapy in order to minimize the risk of developing a peri-implant infection. Further studies are still needed to improve our understanding of all etiological factors in order to better understand prevention and maintenance.

## 5. Conclusions

The present study illustrates the wide range of etiologic factors in peri-implant disease. For prevention, all factors must be considered at every stage of treatment. Adequate pre-surgical and surgical planning is essential to assure prosthodontically optimal implant positioning and minimize risks. Care must be taken to not make surgical, prosthetic, or hygienic compromises that could negatively affect peri-implant health in the long run. The design of the reconstruction should allow for efficient hygiene access for the patient. For patients, especially those with a history of periodontitis who have undergone implant treatment, follow-up maintenance care in addition to monitoring and early detection are needed. 

## Figures and Tables

**Table 1:** Patient and case characteristics. NA: not available.

Gender	Number of Patients (%)
Female	127 (67)
Male	63 (33)
Total	190 (100)
**Age (years)**	
Median	62
Min.	24
Max.	87
**Referral**	**Number of patients (%)**
External	173 (91)
Self/In-house	17 (9)
**Place of implant insertion**	**Number of patients (%)**
Alio loco	86 (45%)
Referral practice	87 (46%)
Center of dental medicine	17 (9%)
**Systemic involvement**	**Number of patients (%)**
Yes	55 (29%)
No	121 (64%)
NA	13 (7%)
**Psychological comorbidity**	**Number of patients (%)**
Present (certified by specialist)	11 (6%)
Not present	163 (86%)
NA	15 (8%)
**Local factors (LFs)**	**Number of patients (%)**
Present	87 (46)
Absent	93 (49)
NA	10 (5)
**Peri-implant keratinized mucosa width**	**Number of patients (%)**
Sufficient	127 (67)
Insufficient	59 (31)
NA	4 (2)
**Risk factors**	**Number of patients (%)**
Present	179 (94)
No clear risk factors	11 (6)

**Table 2:** Prosthetic superstructure and case characteristics. NA: not available.

Cleanability of Prosthetic Reconstruction	Number of Patients (%)
Easy to clean	146 (77)
Moderate	19 (10)
Difficult	18 (9.5)
Impossible to self-clean	3 (1.5)
NA	4 (2)
**Prosthetic reconstruction**	**Number of patients (%)**
Fixed	167 (88)
Removable	19 (10)
NA	4 (2)
**Prosthesis**	**Number of patients (%)**
Cemented	95 (50)
Screwed on	85 (45)
Other	6 (3)
NA	4 (2)
**Prosthetic material**	**Number of patients (%)**
Metal–ceramic restoration	156 (82)
Abutments with bonded ceramic crowns	8 (4)
Other	22 (12)
NA	4 (2)
**Prosthetic reconstruction**	**Number of patients (%)**
Single crown implants	86 (45)
Reconstruction on multiple implants	100 (53)
NA	4 (2)
**Interface between crown and implant on radiograph**	**Number of patients (%)**
Gap-free	86 (45)
Insufficient	100 (53)
NA	4 (2)

**Table 3:** Biological complications and case characteristics. NA: not available.

**Total number of implants**	682
**Number of implants with biological complications**	388
**Diagnosis**	**Number of patients (%)**
Peri-implantitis	158 (83)
Peri-implant mucositis	30 (16)
NA	2 (1)
**Peri-implantitis**	**Number of patients (%)**
Single implant	97 (51)
Multiple implants	93 (49)
**Probing depth Median**	5.5 mm **Mean** 6 mm
**Radiographic form of defect**	**Number of patients (%)**
Horizontal bone loss	84 (44)
Vertical bone loss	59 (31)
Marginal radiolucency	4 (2)
No bone loss visible	38 (20)
NA	5 (3)
**Bone augmentation**	**Number of patients (%)**
Guided bone regeneration/ ridge augmentation	40 (21)
Sinus lift	15 (8)
No augmentation documented	49 (26)
NA	86 (45)
**Overall implant positioning**	**Number of patients (%)**
Optimal implant positioning	152 (80)
Error in implant positioning	17 (19)
NA	2 (1)
**Distance to neighboring structure**	**Number of patients (%)**
Sufficient distance	177 (93)
Insufficient distance	11 (6)
NA	2 (1)
**Relative implant positioning (vertical) Number of patients (%)**	
Appropriate insertion depth	175 (92)
Seated too deeply	13 (7)
NA	2 (1)
**Relative implant positioning**	**Number of patients (%)**
Appropriate buccal-lingual position	177 (93)
Seated too buccally	11 (6)
NA	2 (1)

## Data Availability

Anonymized data can be obtained from the authors.
